# From Referee to Coach: Toward Smart Global Health Regulation

**DOI:** 10.3390/healthcare14213740

**Published:** 2026-11-03

**Authors:** James P. Kingsland, Eddie H. P. Tan, Silke Vogel, John C. W. Lim

**Affiliations:** 1 Digital Clinical Excellence Forum (DiCE), West Kirby CH48 7JA, UK; james@digitalclinicalexcellence.org; 2 School of Medicine, University of Lancashire, Preston PR1 2HE, UK; 3 Centre of Regulatory Excellence, Duke-NUS Medical School, Singapore 169857, Singapore; silke.vogel@duke-nus.edu.sg (S.V.); john.lim@duke-nus.edu.sg (J.C.W.L.)

**Keywords:** smart regulation, health policy, global health, health systems, international regulatory alignment

## Abstract

**Highlights:**

**What are the main findings?**

• This conceptual Perspective proposes an integrative framework for smart health regulation, drawing on established regulatory scholarship, professional experience and illustrative examples rather than empirical validation.
• Safety and innovation should not be treated as automatic opposites; under appropriate conditions, adaptive learning, proportionality, transparent oversight and robust assurance may help align them.

**What are the implications of the main findings?**

• Regulators may need to complement static enforcement with more adaptive regulatory approaches that emphasise learning, proportionality and structured engagement in areas of genuine uncertainty.
• Collaborative regulation may support trust and health-system resilience when combined with transparency, independence and accountability. International alignment should therefore be pursued pragmatically, with attention to context, capacity and equity.

**Abstract:**

Developed from the 2025 Sir Alasdair Breckenridge Lecture, this Perspective draws on selective literature and illustrative examples from the United Kingdom, Singapore, and international frameworks. Adaptive, responsive, and agile approaches to regulation are well established in governance scholarship, yet their application to the combined challenges of health products, digital health-enabled services, and cross-border care remains fragmented. We argue that smart health regulation is best understood not as an entirely new paradigm, but as an integrative framework that synthesises adaptive, collaborative, and learning-oriented approaches for contemporary healthcare systems. The authors propose five practical design principles, four implementation mechanisms and an illustrative Smart Health Regulation Scorecard intended as non-validated heuristic for reflection rather than as a validated instrument or ranking system. The framework is informed by prior regulatory scholarship, comparative practice, the underlying lecture and the authors’ professional experience, and is offered to support discussion on how safety, innovation, accountability, and trust can be balanced across different regulatory settings.

## 1. Background

The Centre of Regulatory Excellence (CoRE) launched an annual lecture series in 2021 in Singapore to honour the memory of the late Professor Sir Alasdair Breckenridge, CoRE’s founding Chair, and foster dialogue on global biomedical and regulatory perspectives, challenges, and advances. The 2025 Sir Alasdair Breckenridge Lecture “From Setbacks to Breakthroughs: The Development of Smart Healthcare Regulation” was delivered by Professor James Kingsland OBE, Clinical Professor in the School of Medicine at the University of Lancashire, Chairperson of the Digital Clinical Excellence Forum in the United Kingdom and member of the Regulatory Advisory Panel with Singapore’s Ministry of Health. The present article is a Perspective developed from that lecture and subsequently refined through selective engagement with relevant literature and illustrative comparative examples.

## 2. Introduction

Healthcare regulation stands at an inflection point. The rapid proliferation of breakthrough technologies, such as advanced therapies, artificial intelligence, digital health platforms, and extended reality applications, has collided with regulatory systems whose fundamental architecture remains rooted in earlier eras, designed for slower-moving, more predictable landscapes. In this paper, healthcare regulation refers primarily to the governance of health products, digital health-enabled services, and selected health-service interfaces where innovation, delivery, and patient protection intersect; it does not attempt a comprehensive legal analysis of every domain of professional regulation or public health law. Existing theories of smart, adaptive, responsive, collaborative, and agile regulation offer important insights, but they are often applied in fragmented ways across these overlapping domains. This creates a conceptual gap when regulators must simultaneously manage technological novelty, cross-border evidence flows, implementation challenges, and public trust. We use the term smart health regulation to describe an integrative governance orientation that is agile yet principled, data-informed yet human-centred, and protective yet enabling.

Yet this moment presents not merely a crisis but an opportunity to reimagine what effective health regulation can be. *Referee regulation*, characterised by adversarial enforcement, rigid rules, and risk aversion, can become misaligned with the pace and character of contemporary healthcare innovation. *Coach-oriented regulation*, emphasising collaborative problem-solving, adaptive frameworks, evidence-based learning, and human-centred design, can be a useful operational orientation in settings characterised by uncertainty and rapid iteration. The challenge is not to replace one role with the other, but to design systems that combine developmental support with independent oversight.

## 3. The Fundamental Challenge: Shifting from Static Compliance to Adaptive Governance

### 3.1. The Regulatory Challenge: Burden, Complexity, and Trust

In some developed regulatory jurisdictions, confidence in regulatory authority may be weakened when operational backlogs, inconsistent assessments, or poorly implemented reforms delay decisions or reduce transparency. Regulators can consequently be perceived as slow or overly burdensome, while innovators may experience complex and duplicative requirements as obstacles to development. These concerns are context-dependent and do not establish a uniform crisis across jurisdictions.

History shows that regulation evolves in response to crisis and learning. To illustrate how these tensions appear in practice, we draw selectively on the United Kingdom and Singapore because they are familiar, well-documented, and institutionally distinct jurisdictions that have both undertaken visible regulatory reform; these examples are illustrative rather than globally representative. In the United Kingdom, a government-commissioned review of the Care Quality Commission (CQC) and a separate Homecare Association report highlighted shortcomings in regulatory performance, illustrating the importance of consistent assessments, effective information systems and transparent oversight [1,2]. Singapore’s system, by contrast, exhibits high capability but increasing density. The Healthcare Services Act (HCSA, 2020) replaced premises-based licensing with a service-based model; the Health Sciences Authority (HSA) applies risk-based approaches to health-product regulation and professional regulatory bodies oversee practice standards. The Licensing Experimentation and Adaptation Programme (LEAP) was introduced as a regulatory sandbox for emerging healthcare service models [3]. Both HSA and the UK Medicines and Healthcare products Regulatory Agency (MHRA) participate in the Access Consortium, which coordinates work-sharing while preserving national decisions [4]. The MHRA also operates an International Recognition Procedure that uses authorisations from specified reference regulators and an AI Airlock sandbox for AI-enabled medical devices [5,6]. Together these examples show both the potential and the governance demands of adaptive approaches. Across both settings, complexity is not a synonym for quality, and inspection alone is unlikely to deliver continuous improvement in rapidly evolving environments.

### 3.2. Fragmentation as a Global Risk

Regulatory fragmentation across jurisdictions can require duplicative reviews and contribute to differences in approval timing and patient access, although these outcomes are also shaped by pricing, reimbursement, manufacturing capacity, commercial strategy, and health-system infrastructure [7,8]. Differences in regulatory definitions, timelines, evidence expectations and post-approval requirements can make it harder to plan coherent development strategies for innovative therapies. Manufacturers may still face unique registration requirements, country-specific clinical trial mandates, in-country lot testing, country-specific labelling, and prior-approval expectations in countries of origin or manufacture. Each divergence may necessitate separate submissions, additional evidence generation and incremental cost. For ultra-rare diseases with limited patient populations, such frictions can weaken the commercial feasibility of global development and delay patient access.

The implications may be especially severe for lower- and middle-income countries. Where regulatory frameworks diverge substantially, resource-constrained authorities may be less able to leverage work already performed by larger agencies and may need to duplicate technically demanding assessments with limited capacity. WHO’s good reliance practices offer one response by encouraging authorities to use trusted regulatory work while retaining responsibility for national decisions [7]. Manufacturers, responding rationally to fragmentation and asymmetric market opportunity, may prioritise affluent settings with clearer or faster pathways. At the same time, access disparities are mediated not only by regulation but also by financing, procurement, logistics, manufacturing scale, and health-system readiness. Smart health regulation should therefore be understood as one component of a broader access ecosystem rather than a standalone solution to inequity.

The World Health Organisation (WHO) International Health Regulations (IHR) [9], the International Council for Harmonisation of Technical Requirements for Pharmaceuticals for Human Use (ICH) [10], and the International Medical Device Regulators Forum (IMDRF) [11] provide important coordination architectures, although their authority, scope and enforceability differ. The COVID-19 pandemic highlighted global supply-chain vulnerabilities and inequities in access [12]. Despite these architectures, sovereignty, institutional history, confidentiality constraints and uneven capacity help sustain a patchwork of health regulations. The result can be delay, duplication, and avoidable inequity, even where the policy intent of individual regulators is sound.

### 3.3. The Conceptual Foundation: Regulation’s Dual Role

A critical insight, articulated across multiple regulatory jurisdictions and supported by contemporary governance scholarship, is that regulation serves both protective and enabling functions. Traditional regulatory frameworks emphasise protection through rule-setting, monitoring and sanctioning, reflecting legitimate historical lessons from harms associated with medicines and medical products [13]. Yet regulatory science increasingly recognises that regulation also helps shape innovation by clarifying evidentiary expectations, signalling priorities, supporting safer development pathways, and creating the institutional conditions within which beneficial innovation can emerge [14].

Regulation therefore operates through multiple mechanisms, not only enforcement but also standard-setting, priority-signalling, capacity-building, and innovation guidance. Its objectives also extend beyond safety alone. While patient safety remains paramount, regulators increasingly confront adjacent concerns including affordability, sustainability, environmental impact, data privacy, cybersecurity, intellectual property, and equity of access. When such secondary objectives are not made explicit, they may still influence decisions implicitly, contributing to inconsistency and unintended consequences. A smarter regulatory approach should make these trade-offs more visible, governable, and accountable.

The COVID-19 pandemic highlighted the need for adaptive regulatory approaches including expedited pathways and intensive sponsor-regulator dialogue. Such approaches must be considered alongside the exceptional legal authorities, resources and surveillance available during an emergency [15]. Regulatory divergence, unequal access and communication challenges remained important concerns [16]. The broader lesson is therefore not that speed is inherently cost-free, but that innovation, transparency, institutional independence, and post-market monitoring must be designed together if trust is to be sustained [17].

## 4. The Referee vs. Coach Paradigm: Operationalising Transformation Towards Smart Regulation

### 4.1. Regulatory Roles: From Enforcement to Partnership

Regulators may need to move beyond solely static enforcement (“referee”) towards iterative policy development, regulatory learning through experimentation, and stakeholder co-creation (“coach”) in areas of genuine uncertainty [18,19]. We use the metaphor of “referee” and “coach” as a heuristic to distinguish operational orientations, not as an absolute dichotomy.

A “referee” operates through rule enforcement: establishing the rules of play, monitoring for violations, and penalising deviations. This role remains essential in domains with mature evidence standards, stable technologies, wilful non-compliance, fraud, repeated safety failures, or predictable risks. It provides clarity, deterrence, and accountability, but can become limiting if applied without adaptation to genuinely novel technologies and uncertain environments.

In contrast, a “coach” operates through partnership and learning: establishing principles and high standards while working with participants to navigate uncertainty, improve performance, and achieve shared public health objectives. Applied to healthcare regulation, this may include early scientific advice, sandbox supervision, dialogue-centred problem-solving, principles-based expectations, and greater transparency of regulatory reasoning to inform better products and services. Such coaching is most appropriate where uncertainty is real, evidence pathways are still emerging, and regulators can add value without compromising independence.

Critically, the coach-oriented model does not eliminate the need for enforcement or penalty. Instead, it recalibrates when and how developmental support is used alongside assurance. Collaborative approaches require safeguards against regulatory capture, conflicts of interest, unequal access to advice, and erosion of institutional independence. Assurance and improvement functions may therefore need clear governance separation, transparent documentation, conflict-management processes, and auditable decision trails. The key design task is to allow learning and dialogue without blurring accountability.

### 4.2. Defining Smart Regulation

Smart regulation has long been embedded in policy discourse as a regulatory approach emphasising flexibility, innovation, and multi-stakeholder participation to enhance policy and legislative effectiveness. Originating with Gunningham and colleagues, smart regulation emphasises complementary policy instruments and escalating responses that encourage actors to exceed baseline compliance [20]. The European Commission and OECD later expanded this agenda towards adaptive, learning-based governance and broader stakeholder engagement [21,22,23]. Related scholarship on agile and anticipatory governance likewise highlights regulatory foresight, experimentation, and learning in the face of rapid technological change [18,19].

Our contribution is therefore not to claim novelty for adaptiveness itself, but to integrate these strands for contemporary healthcare systems in which health products, digital technologies, service delivery, and cross-border evidence increasingly interact. In this paper, smart health regulation refers to a healthcare-specific synthesis of these traditions. We distinguish it from adjacent concepts not by wholly separate principles, but by its explicit focus on combining protective and enabling functions across the lifecycle of innovation, implementation, and post-market learning.

The five design principles below were developed through an interpretive synthesis of the literature cited above, illustrative comparative practice examples, the underlying Breckenridge Lecture, and the authors’ professional experience in health regulation. They are intended as a conceptual framework for analysis and dialogue rather than as a definitive taxonomy. In our view, smart health regulation aims for clarity over complexity and learning over blame (Table 1).

Taken together, these principles seek to convert episodic inspection into continuous feedback and to frame regulation as a learning system while remaining accountable for safety, transparency, and equitable adoption.

### 4.3. Designing Operational Pathways for Smart Regulation

Smart regulation requires operationalisation through multiple dimensions: horizon scanning to anticipate emerging technologies; regulatory sandboxes to enable controlled pilot testing; lifecycle assessment to support ongoing post-market evaluation; stakeholder engagement to maintain structured dialogue with innovators, clinicians, patients, and communities; transparent decision rationales to strengthen trust and consistency; and performance metrics that look beyond compliance alone towards safety, timeliness, equity, and institutional trust. The feasibility of these mechanisms, however, depends on regulatory capacity, data infrastructure, technical expertise, governance safeguards, sustainable resources, and legal authority, all of which vary substantially across jurisdictions, especially in lower-resource settings.

We highlight four implementation mechanisms that can help agencies operationalise smart health regulation in practice (Table 2).

The overall architecture is sequential. The five design principles define what smart health regulation seeks to achieve; the four implementation mechanisms describe how agencies may operationalise those principles; the scorecard offers illustrative domains for monitoring progress; and the international initiatives indicate where cross-border coordination may add value.

### 4.4. Measuring Progress in Smart Health Regulation Adoption

Performance metrics evaluate how well regulations achieve their intended outcomes, rather than merely measuring adherence to rules. We therefore propose an illustrative Smart Health Regulation Scorecard that could support structured self-assessment or peer discussion across five domains (Table 3). Its default unit of assessment is the regulatory agency, applied through agency self-assessment or peer review. It could also be used for a defined pathway or product class, provided that the assessor and scope are stated explicitly so that the 0–2 criteria are interpreted consistently. The scorecard is intentionally unweighted and non-validated; it is not presented as a ranking tool, but as a heuristic that would require piloting, inter-rater testing, and refinement before any formal benchmarking use. The illustrative descriptors should not be interpreted as validated measurements, combined into an overall performance score or used to rank agencies or jurisdictions.

### 4.5. International Alignment

We further suggest that an observatory, linked to an international organisation (e.g., the World Health Organization or OECD), could maintain comparative dashboards to support peer learning, transparency, and diffusion. Any such observatory would need clear governance, agreed data standards, and careful management of confidentiality, legal, and political sensitivities.

Achieving greater international convergence requires sustained political and institutional effort rather than assuming immediate global harmonisation. We therefore outline four strategic initiatives. First, an International Healthcare Regulatory Framework (IHRF) could articulate a global charter of minimum expectations for safety, ethics, transparency, and digital oversight. Second, a Global Evidence Platform could enable federated, privacy-preserving exchange of clinical trials and post-market evidence. Third, aligned rapid adoption pathways could use pre-agreed templates to facilitate cross-jurisdictional uptake of validated innovations. Fourth, targeted capacity building could support more equitable participation by lower- and middle-income regulators in harmonisation efforts. These options would still need to accommodate differences in legal systems, political priorities, digital maturity, confidentiality rules, and resource constraints; they should therefore be understood as directional proposals rather than immediate universal solutions.

## 5. Limitations

This Perspective has several limitations. It was developed from a single lecture and uses an interpretive rather than systematic synthesis of selected literature. The United Kingdom and Singapore were purposively selected as familiar, well-documented examples and are not globally representative. The proposed scorecard is conceptual and non-validated. The authors’ institutional positions may also shape interpretation: CoRE organises the lecture series and is an affiliation of the authors, and James P. Kingsland serves on the Regulatory Advisory Panel of Singapore’s Ministry of Health, whose regulatory system is discussed. These limitations reinforce that the framework is a heuristic agenda for testing and adaptation rather than a generalisable model.

## 6. Conclusions

Smart health regulation depends as much on culture as on code. It demands leadership that is firm enough to uphold standards, reflective enough to recognise uncertainty, and constructive enough to support improvement. The late Sir Alasdair Breckenridge exemplified this balance: rigour with humility, standards with compassion [25]. The objective is not bureaucratic control alone, but durable public trust that innovation will be evaluated and implemented as safely, transparently, and fairly as circumstances allow.

Isolated national regulation is becoming harder to sustain in areas characterised by shared technologies, transnational data and global supply chains. Safety and innovation should not be treated as automatic opposites; under the right institutional conditions, they can be better aligned through intelligent governance. Population health increasingly depends on coherence, clarity, and collective learning. Regulators who act only as referees may struggle in such settings, whereas those able to combine referee and coach functions, with appropriate safeguards, may be better positioned to respond.

Established coordination and work-sharing initiatives show that collective governance in healthcare is possible, although the field presents distinctive legal, ethical, and political complexities [4,7,10,11]. Many of the tools for smarter regulation are already visible, but their adoption will depend on evidence, institutional capacity, and trust. Smart health regulation should therefore be seen less as a universal prescription than as a practical agenda for continued experimentation, evaluation, and international dialogue.

## Figures and Tables

**Table 1:** Five principles of smart health regulation.

Clarity and transparency	Rules, rationales, decision criteria, and where feasible, data are communicated in forms accessible to stakeholders.
Risk-proportionate oversight	Evidence requirements and review intensity are calibrated to risk, uncertainty, and post-market monitoring capacity.
Separation of roles with shared data	Assurance and improvement functions remain institutionally distinct but can learn from shared, well-governed data.
Continuous learning	Approval, licensing, or service authorisation marks the start of ongoing evidence generation and regulatory adaptation.
Adoption and diffusion as outcomes	Regulators and health systems consider not only approval, but also uptake, equitable access, and implementation of proven innovations.

**Table 2:** Four implementation mechanisms for smart health regulation.

1. Regulatory sandboxes with exit rules Regulatory sandboxes can support controlled experimentation when pilots are transparent, time-limited, and linked to explicit learning objectives, monitoring arrangements and exit criteria. Singapore’s LEAP and the MHRA AI Airlock illustrate sandbox approaches in healthcare services and AI-enabled medical devices, respectively [3,5]. The US Food and Drug Administration’s Digital Health Software Precertification Pilot also illustrates the importance of evaluating the feasibility of proposed regulatory models and defining learning and exit decisions [24].
2. Reliance and mutual recognition WHO good reliance practices support the use of trusted regulatory work while preserving the relying authority’s independence and accountability [7]. The Access Consortium, which includes both HSA and MHRA, coordinates work-sharing, while the MHRA International Recognition Procedure uses authorisations from specified regulators [4,6]. These approaches still depend on legal compatibility, local pharmacovigilance, transparency about evidentiary assumptions and confidence in the originating review.
*** ***3. Federated evidence sharing A federated data model—local ownership, common metadata, secure analytics—allows safety signals and benefit-risk information to circulate without requiring unrestricted data pooling. Such models, however, require governance rules for privacy, cybersecurity, consent, interoperability, and access.
4. Culture of diffusion Health systems often mistake innovation for invention. Real innovation is invention plus adoption. Policy incentives should therefore reward timely and equitable implementation of validated technologies while recognising that diffusion depends on workforce readiness, financing, procurement, and service redesign.

Abbreviations: HSA, Health Sciences Authority; LEAP, Licensing Experimentation and Adaptation Programme; MHRA, Medicines and Healthcare products Regulatory Agency; WHO, World Health Organization.

**Table 3:** Example domains for the Smart Health Regulation Scorecard.

Domains	Illustrative Descriptors (0–2) (0 = Absent/Opaque; 1 = Partial or Pilot Implementation; 2 = Routine and Publicly Evidenced)
Publication of plain-language regulatory decisions	0 = no accessible summaries; 1 = selective or inconsistent summaries; 2 = routine public summaries with decision rationale.
2. Risk-proportionate linkage between risk and evidence requirements	0 = little explicit proportionality; 1 = proportionality used in some pathways; 2 = clearly documented, consistently applied proportionality.
3. Structural separation of assurance and improvement functions	0 = no clear separation; 1 = partial separation or ad hoc governance; 2 = explicit separation with documented interfaces and accountability.
4. Post-market evidence and surveillance expectations	0 = limited expectations; 1 = applied to selected products or pilots; 2 = routine lifecycle evidence plans with active surveillance.
5. Public reporting of adoption, equity, or diffusion indicators	0 = not reported; 1 = internal or irregular reporting; 2 = regular public reporting linked to implementation outcomes.

Note: This scorecard is an illustrative, non-validated heuristic for structured reflection and discussion. Its descriptors are not validated measurements and should not be used to generate overall scores or rankings.

## Data Availability

No new data were created or analysed in this study. Data sharing is not applicable to this article.

## Abbreviations

The following abbreviations are used in this manuscript:

CoRE	Centre of Regulatory Excellence
CQC	Care Quality Commission
HCSA	Healthcare Services Act
HSA	Health Sciences Authority
ICH	International Council for Harmonisation of Technical Requirements for Pharmaceuticals for Human Use
IHR	International Health Regulations
IHRF	International Healthcare Regulatory Framework
IMDRF	International Medical Device Regulators Forum
LEAP	Licensing Experimentation and Adaptation Programme
MHRA	Medicines and Healthcare products Regulatory Agency
OECD	The Organisation for Economic Co-operation and Development
WHO	World Health Organisation
